# Altered Na/Ca exchange distribution in ventricular myocytes from failing hearts

**DOI:** 10.1152/ajpheart.00597.2015

**Published:** 2015-11-13

**Authors:** Hanne C. Gadeberg, Simon M. Bryant, Andrew F. James, Clive H. Orchard

**Affiliations:** School of Physiology, Pharmacology, and Neuroscience, University of Bristol, Bristol, United Kingdom

**Keywords:** cardiac myocytes, T tubules, intracellular Ca, heart failure

## Abstract

In mammalian cardiac ventricular myocytes, Ca efflux via Na/Ca exchange (NCX) occurs predominantly at T tubules. Heart failure is associated with disrupted t-tubular structure, but its effect on t-tubular function is less clear. We therefore investigated t-tubular NCX activity in ventricular myocytes isolated from rat hearts ∼18 wk after coronary artery ligation (CAL) or corresponding sham operation (Sham). NCX current (*I*_NCX_) and l-type Ca current (*I*_Ca_) were recorded using the whole cell, voltage-clamp technique in intact and detubulated (DT) myocytes; intracellular free Ca concentration ([Ca]_i_) was monitored simultaneously using fluo-4. *I*_NCX_ was activated and measured during application of caffeine to release Ca from sarcoplasmic reticulum (SR). Whole cell *I*_NCX_ was not significantly different in Sham and CAL myocytes and occurred predominantly in the T tubules in Sham myocytes. CAL was associated with redistribution of *I*_NCX_ and *I*_Ca_ away from the T tubules to the cell surface and an increase in t-tubular *I*_NCX_/*I*_Ca_ density from 0.12 in Sham to 0.30 in CAL myocytes. The decrease in t-tubular *I*_NCX_ in CAL myocytes was accompanied by an increase in the fraction of Ca sequestered by SR. However, SR Ca content was not significantly different in Sham, Sham DT, and CAL myocytes but was significantly increased by DT of CAL myocytes. In Sham myocytes, there was hysteresis between *I*_NCX_ and [Ca]_i_, which was absent in DT Sham but present in CAL and DT CAL myocytes. These data suggest altered distribution of NCX in CAL myocytes.

## Abstract (precis)

Na/Ca exchange (NCX) is normally located predominantly in the T tubules of cardiac ventricular myocytes. However, redistribution of NCX occurs in myocytes from failing hearts, resulting in more uniform distribution between T tubule and surface sarcolemma; this alters access of NCX to Ca released from sarcoplasmic reticulum and thus cellular Ca handling.

## NEW & NOTEWORTHY

*Na/Ca exchange (NCX) is normally located predominantly in the T tubules of cardiac ventricular myocytes. However, redistribution of NCX occurs in myocytes from failing hearts, resulting in more uniform distribution between T tubule and surface sarcolemma; this alters access of NCX to Ca released from sarcoplasmic reticulum and thus cellular Ca handling*.

contraction of cardiac ventricular myocytes is initiated by Ca influx across the cell membrane via the L-type Ca current (*I*_Ca_), which activates ryanodine receptors (RyRs) in adjacent sarcoplasmic reticulum (SR) membrane, triggering Ca-induced Ca release (CICR) from the SR. Relaxation occurs as a result of Ca removal from the cytoplasm into the SR, via a Ca ATPase (SERCA), and across the cell membrane, predominantly via Na/Ca exchange (NCX) (2).

In mammalian ventricular myocytes, *I*_Ca_, CICR, and Ca efflux via NCX occur predominantly at invaginations of the surface membrane, called T tubules (5). Presumably because of this colocation, Ca close to the site of CICR appears to be more effective than bulk cytoplasmic Ca at stimulating Ca efflux via NCX, and thus–since NCX carries three Na for each Ca–the associated inward (depolarizing) NCX current (*I*_NCX_) (35). This is important not only for normal Ca efflux but also because under conditions of Ca overload, spontaneous SR Ca release occurs, activating inward *I*_NCX_ and causing delayed afterdepolarisations (DADs), which can trigger action potentials and arrhythmias (18). Because of the proximity of RyRs and NCX, it seems likely that such activity will arise predominantly at T tubules.

Heart failure (HF) is associated with disruption of the t-tubular network (21, 22) and redistribution of *I*_Ca_ from the T tubules to the surface membrane, although RyR distribution appears unaltered (7). However, the effect of HF on the distribution of *I*_NCX_ is unknown but is important because changes may alter the proximity of NCX to the site of CICR, and thus *I*_NCX_ and Ca efflux. In the present study we therefore investigated the distribution of *I*_NCX_ between the t-tubular and surface membranes, and its functional consequences, in myocytes from normal and HF rats.

## MATERIALS AND METHODS

### 

#### Surgical model of HF.

Ligation of the left anterior descending coronary artery of adult male Wistar rats (CAL) was performed as previously described (7). The corresponding sham operation (Sham) was identical except that no tie was placed around the coronary artery. All procedures were performed in accordance with UK legislation and approved by the University of Bristol Ethics Committee. As reported in another study using cells from these animals, CAL had no significant effect on body weight or tibia length, but significantly increased heart and lung weights relative to body weight and tibia length, decreased ejection fraction, and increased left ventricular diastolic and systolic volumes, indicative of early stage HF (7).

#### Myocyte isolation and detubulation.

Myocytes were isolated from the left ventricular free wall and septum of Sham and CAL animals 18.6 ± 0.3 and 18.5 ± 0.3 wk after surgery, respectively, as previously described (7) and stored for 2–8 h before use on the day of isolation. Myocyte detubulation (DT), physical and functional uncoupling of the T tubules from the surface membrane, was achieved using formamide-induced osmotic shock, as previously described (4, 6, 7).

#### Solutions.

All reagents were obtained from Sigma-Aldrich (Poole, UK) unless otherwise specified. Cells were superfused with solution containing (in mmol/l) 133 NaCl, 1 MgSO_4_, 1 CaCl_2_, 1 Na_2_HPO_4_, 10 d-glucose, and 10 HEPES (pH 7.4, NaOH); 5 CsCl was added to inhibit K currents. The pipette solution contained (in mmol/l) 110 CsCl, 20 tetraethylammonium chloride, 0.5 MgCl_2_, 5 MgATP, 10 HEPES, 0.4 GTP-Tris (pH 7.2, CsOH), and 0.1 pentapotassium salt of the fluorescent Ca indicator fluo-4 (Life Technologies, Paisley, UK).

#### Measurement of I_Ca_ and I_NCX._

Myocytes were placed in a chamber mounted on a Diaphot inverted microscope (Nikon UK, Kingston-upon-Thames, UK). Membrane currents and cell capacitance were recorded with the whole cell patch-clamp technique using an Axopatch 200B patch clamp amplifier, a Digidata 1440A analog-to-digital converter, and pClamp 10 software (Molecular Devices, Reading, UK), which was used for data acquisition (at 2 kHz) and analysis. Pipette tip resistances were typically 1.5–3.0 MΩ when filled with pipette solution.

Holding potential was −80 mV; a 300-ms ramp to −40 mV was used to inactivate *I*_Na_, followed by step depolarization to 0 mV for 300 ms to activate *I*_Ca_, at a frequency of 1 Hz. *I*_Ca_ was measured as the difference between peak inward current and current at the end of the pulse to 0 mV. Once steady-state was achieved, stimulation was stopped, and after 10-s quiescence, caffeine (10 mmol/l) was rapidly applied to the cell to cause spatially and temporally uniform release of SR Ca (3); the resulting inward current due to Ca extrusion via NCX was recorded at −80 mV. Following wash-off of caffeine, stimulation was restarted and continued until a steady-state was reached. The protocol was then repeated, but NiCl_2_ (10 mmol/l) was applied 10 s before application of caffeine in the continued presence of Ni to inhibit NCX.

Membrane currents were normalized to membrane capacitance (a function of membrane area) to give current density. The distribution of membrane current between the surface and t-tubular membranes was calculated from the currents measured in intact (whole cell) and DT (surface membrane only) myocytes, as previously described (7).

#### Measurement of intracellular Ca.

Fluo-4 fluorescence was excited at 450–488 nm and emitted fluorescence collected at wavelengths > 560 nm. Normalized fluo-4 fluorescence (F/F_0_) was converted to intracellular free Ca concentration ([Ca]_i_) as follows:

(1) $$[\text{Ca}{]}_{\text{i}}=\frac{K_{\text{d}}F/F_{0}}{\frac{K_{\text{d}}}{{\left[\text{Ca}\right]}_{\text{rest}}}-F/F_{0}+1}$$

where resting Ca ([Ca]_rest_) was assumed to be 0.1 μmol/l and the dissociation constant (*K*_d_) for fluo-4 in situ was 1.1 μmol/l (8, 20).

The rate of decay of Ca transients was obtained by fitting single exponential functions to the declining phase of the *I*_Ca_- and caffeine-induced Ca transients. The fitted rate constants (*k′*) were multiplied by the appropriate (Sham or CAL) buffering power (calculated by plotting [Ca]_i_ against [Ca]_total_, as previously described) (12, 33) to correct for the effect of buffering power and thus allow comparison of the rate of Ca extrusion in Sham and CAL myocytes. This corrected rate constant (*k*) during application of caffeine (*k*_Caff_) was used as an index of the rate of total sarcolemmal Ca efflux, and that in the presence of caffeine plus Ni (*k*_Ni_) as the rate of the slow (non-SR, non-NCX) Ca extrusion pathways. The rate of Ca removal via NCX (*k*_NCX_) was calculated as *k*_Caff_-*k*_Ni_, and the rate of Ca uptake by SR (*k*_SR_) was calculated as the difference between the rate of decline of the *I*_Ca_-induced Ca transient and *k*_Caff_. These rate constants were also used to calculate the percent contribution of these pathways to Ca removal from the cell cytoplasm, as previously described (25, 36).

#### Statistical analysis.

Data are expressed as means ± SE. The errors of derived variables and the subsequent statistical analysis were calculated using propagation of errors from the constituent measurements. Student's *t*-tests and 2-way ANOVA with the Bonferroni post hoc test were used as appropriate. Statistical significance was taken as *P* < 0.05. All statistical tests were performed on the number of cells. Sample sizes (*n*) are given as *c*/*h*, where *c* is the number of cells used from *h* hearts.

## RESULTS

### 

#### The effect of CAL on NCX distribution.

Cell capacitance (a function of membrane area) was significantly larger in CAL myocytes [240.2 ± 20.8 pF (Sham) vs. 375.0 ± 63.0 pF (CAL); *n* = 12/6 and 8/4, respectively; *P* = 0.004]; this was accompanied by a nonsignificant increase in cell volume [33.5 ± 5.1 pl (Sham) vs. 49.0+6.1 pl (CAL)], resulting in no significant difference in cell surface area-to-volume ratio, as previously reported during cellular hypertrophy (15, 29).

**Fig. 1.**
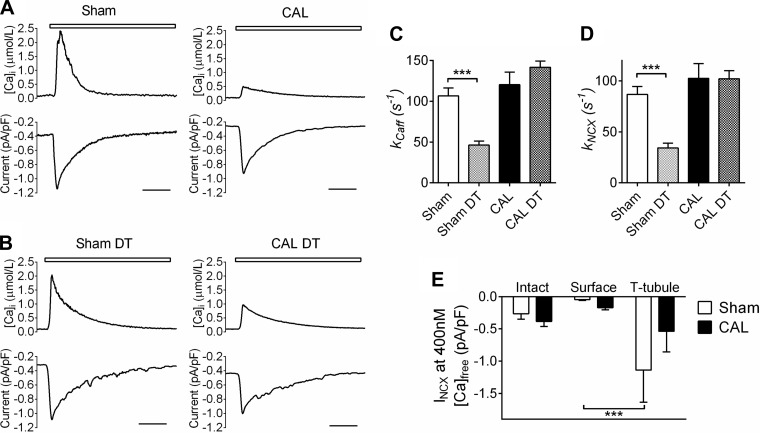
*A*: representative records of intracellular free Ca concentration ([Ca]_i_) and the associated inward Na/Ca exchange (NCX) current (*I*_NCX_) during the application of 10 mM caffeine (white bar) in sham operation (Sham) and coronary artery ligation (CAL) cells. Scale bars represent 2 s. *B*: representative records of [Ca]_i_ and the associated inward current (*I*_NCX_) during the application of 10 mM caffeine (white bar) in detubulated (DT) Sham and CAL cells. Scale bars represent 2 s. *C*: mean rate constants for the decline of the caffeine-induced Ca transient (*k*_Caff_); *n* = 12/6, 11/5, 8/4, 7/4 for Sham, Sham DT, CAL, and CAL DT, respectively. *D*: mean *k*_NCX_; *n* = 12/6, 11/5, 8/4, 5/3 for Sham, Sham DT, CAL, and CAL DT, respectively. *E*: *I*_NCX_ density in the whole cell and at the surface and t-tubular membranes, determined at 400 nM [Ca]_i_ during the declining phase of the caffeine transient. ***P < 0.001 with Bonferroni posttest.

Figure 1*A* shows representative caffeine-induced Ca transients (*top*) and accompanying membrane currents (*bottom*) recorded from Sham and CAL myocytes. Caffeine-induced Ca transient amplitude was significantly smaller in CAL compared with Sham myocytes [2.42 ± 0.63 μM (Sham) vs. 0.78 ± 0.44 μM (CAL); *P* < 0.05] and buffering power, assessed as described in the materials and methods, was significantly larger in CAL myocytes [134 ± 66 (Sham) vs. 294 ± 46 (CAL); *P* < 0.01]. Representative caffeine-induced Ca transients and *I*_NCX_ in DT Sham and CAL cells are shown in Fig. 1*B*: DT did not significantly alter peak [Ca]_i_ in Sham cells (2.06 ± 0.69 μM) but increased peak [Ca]_i_ in CAL cells (to 2.30 ± 1.09 μM).

In Sham myocytes, the decline of the caffeine-induced Ca transient, corrected for buffering power (see materials and methods), was significantly slowed by DT [*k*_Caff_, 106.8 ± 9.5 (Sham) vs. 46.3 ± 5.0 s^−1^ (Sham DT); *P* < 0.001; Fig. 1*C*], consistent with loss of t-tubular Ca efflux pathways. In CAL myocytes, the decline of the caffeine-induced Ca transient was not significantly different from Sham myocytes and not significantly altered by DT [*k*_Caff_, 120.4 ± 15.2 (CAL) vs. 141.8 ± 7.7 s^−1^ (CAL DT); Fig. 1*C*], consistent with reduced t-tubular sarcolemmal Ca efflux in CAL myocytes.

To investigate the role of NCX in these changes, exposure to caffeine was repeated in the presence of Ni to inhibit NCX. Figure 1*D* shows the rate of Ca extrusion via NCX (*k*_NCX_); DT significantly decreased *k*_NCX_ in Sham cells [86.8 ± 7.8 (Sham) vs. 34.3 ± 4.8 s^−1^ (Sham DT); *P* < 0.0001], compatible with loss of t-tubular NCX. In CAL myocytes, *k*_NCX_ was not significantly different from Sham myocytes and not significantly altered by DT [102.5 ± 14.5 (CAL) vs. 102.2 ± 8.0 s^−1^ (CAL DT)], implying that although the rate of Ca extrusion via NCX is similar in Sham and CAL myocytes, there is little Ca extrusion via t-tubular NCX in these cells.

We used *I*_NCX_ in intact and DT myocytes to quantify its distribution between the surface and t-tubular membranes. Since NCX activity depends on Ca adjacent to the exchanger, we measured *I*_NCX_ at a [Ca]_i_ of 400 nmol/l during the descending phase of the caffeine transient, when Ca has been shown to be uniformly distributed throughout the cytoplasm in both intact and DT cells (3). Figure 1*E* shows that whole cell *I*_NCX_ density determined in this way was not significantly different in Sham and CAL myocytes and that in Sham myocytes, *I*_NCX_ density is significantly greater in the t-tubular membrane than at the cell surface, as previously reported (14), resulting in a T tubule-to-surface sarcolemma *I*_NCX_ ratio of 25:1. However, the distribution of *I*_NCX_ is different in CAL myocytes, decreasing at the T tubules by ∼50% and increasing at the surface membrane by ∼300%, resulting in no significant difference in *I*_NCX_ density between the two membranes in these cells and an *I*_NCX_ T tubule-to-surface sarcolemma ratio of 3:1. This suggests that the slower Ca extrusion via NCX following DT of Sham cells is due to loss of t-tubular NCX and that the lack of effect of DT on the rate of Ca extrusion via NCX in CAL cells is due to its relocation away from T tubules.

#### The effect of CAL on cellular Ca handling.

The preceding data show redistribution of *I*_NCX_ in CAL myocytes. NCX is one of the major Ca efflux pathways that compete for cytoplasmic Ca (1, 25), so that a decrease of NCX activity at the T tubules (the site of CICR and SERCA) (24, 32) might alter the balance of Ca removal via NCX and SR (30), thereby altering SR Ca content and thus Ca release and *I*_NCX_. We therefore determined the effect of CAL on the contribution of different pathways to Ca removal.

**Fig. 2.**
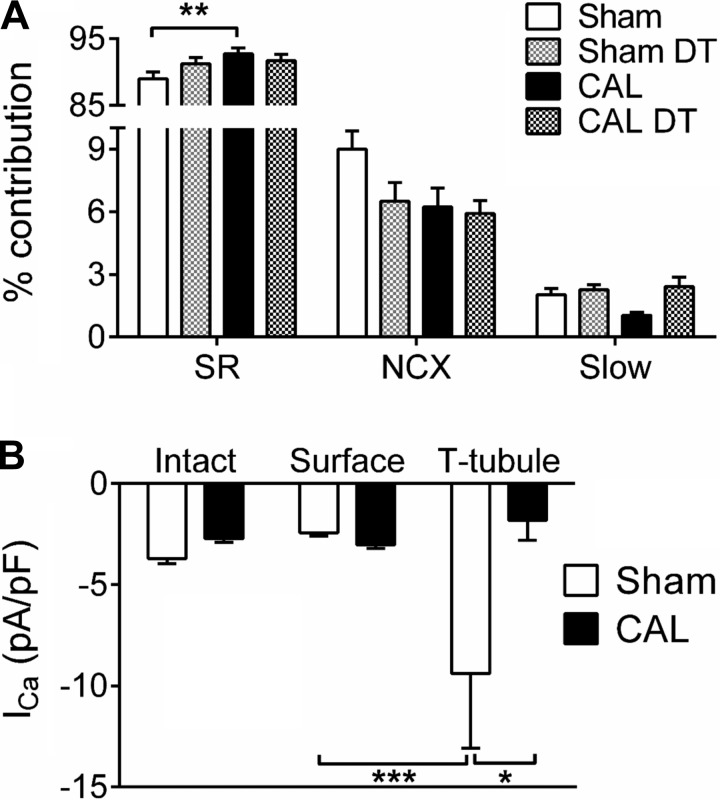
*A*: mean percent contribution of different pathways to Ca removal from the cytoplasm of Sham, DT Sham, CAL, and DT CAL cells; *n* = 12/6, 11/5, 8/4, 5/3, respectively. *B*: distribution of Ca current (*I*_Ca_) density between the surface and t-tubular membranes; *n* = 12/6 and 8/4 for Sham and CAL, respectively; only statistical comparisons between t-tubular and surface membranes are shown. *P < 0.05, **P < 0.01, and ***P < 0.001 with Bonferroni posttest.

Figure 2*A* shows the percent contribution of different pathways to Ca removal from the cytoplasm. There was no significant difference in the contribution to Ca removal by the “slow” Ca extrusion pathways (sarcolemmal Ca ATPase and mitochondria) between the four groups. In Sham myocytes, DT caused a small decrease in the fraction of Ca removed via NCX and increase in Ca removal by the SR, consistent with loss of NCX following DT. In CAL myocytes, the fraction of Ca removed via NCX was smaller than in Sham cells, despite no significant change in whole cell *I*_NCX_ density at a given free [Ca]_i_ (Fig. 1*E*); this was accompanied by a significant increase in the fraction of Ca removed by SR. DT of CAL myocytes had no significant effect on the fraction of Ca removed via NCX or SR, consistent with less t-tubular NCX activity in these cells. These data suggest decreased access of NCX to Ca ions, and thus Ca efflux, in CAL compared with Sham myocytes, presumably as a result of its change in location away from the site of SR Ca release at the T tubules, possibly exacerbated by decreased Ca release at the T tubules as a result of redistribution of *I*_Ca_ and altered t-tubular morphology (7, 21). They also suggest that this redistribution leads to an increase in fractional SR Ca uptake, which would be expected to increase SR Ca release, given its steep dependence on SR Ca content (26, 34).

To determine whether the changes in NCX distribution were quantitatively different from the redistribution of *I*_Ca_ previously reported (7), which might alter Ca balance across the t-tubular membrane and thus the effect of loss of T tubules during HF on cellular Ca balance, we calculated *I*_Ca_ distribution in Sham and CAL myocytes. We have previously reported changes in *I*_Ca_ distribution in CAL myocytes (7) when Ca was buffered using BAPTA in the patch pipette. It was therefore possible that the redistribution of *I*_Ca_ might differ under the present conditions in which no exogenous Ca buffer apart from fluo-4 was present. However, in agreement with previous work, DT of Sham myocytes decreased *I*_Ca_ density from −3.71 ± 0.25 to −2.70 ± 0.25 pA/pF, whereas DT of CAL myocytes had no significant effect on *I*_Ca_ density [−2.71 ± 0.19 (CAL) vs. −2.93 ± 0.21 pA/pF (CAL DT)]. These data were used to calculate the distribution of *I*_Ca_ between the t-tubular and surface membranes, as previously described (7). Figure 2*B* shows that consistent with previous work (7), *I*_Ca_ density was significantly higher in the T tubules of Sham myocytes than in their surface membrane and that in CAL myocytes, t-tubular *I*_Ca_ density was significantly smaller than in Sham myocytes [t-tubular *I*_Ca_, −9.39 ± 3.70 (Sham) vs. −1.80 ± 0.99 pA/pF (CAL); *n* = 12/6 and 8/4, respectively; *P* < 0.05] and not significantly different from *I*_Ca_ density in the surface membrane. Since CAL has little effect of the rate of inactivation of *I*_Ca_ at the t-tubular or surface membranes (7), Ca influx via *I*_Ca_ will reflect these changes in current density.

The ratio of t-tubular *I*_NCX_ to *I*_Ca_ density obtained from these data is 0.12 in Sham myocytes and 0.30 in CAL myocytes. Thus t-tubular density of *I*_NCX_ relative to *I*_Ca_ is greater in CAL than in Sham myocytes; this will result in DT causing greater loss of NCX (and thus Ca efflux) relative to *I*_Ca_ (and thus Ca influx) in CAL cells, which would be expected to increase cellular Ca loading. This is consistent with the observation that SR Ca content was not significantly affected by DT in Sham myocytes but was significantly increased (*P* < 0.05) by DT in CAL myocytes [SR Ca content, 78.8 ± 8.2 μM (Sham); 63.3 ± 9.8 μM (Sham DT); 58.7 ± 5.8 μM (CAL); and 105.4 ± 17.4 μM (CAL DT)].

#### The effect of CAL on the relationship between [Ca]_i_ and I_NCX_.

The data above suggest that decreased t-tubular NCX in CAL myocytes decreases access of NCX to Ca released from the SR, thereby increasing fractional SR Ca uptake; they also suggest that SR Ca load might be increased following loss of T tubules, as a result of the relative changes in Ca influx and efflux. The consequent increase in SR Ca content will alter NCX activity by altering release. However, *I*_NCX_ activity may also be altered directly as a result of the change in the colocation of NCX and RyRs.

Previous work has shown hysteresis in the relationship between bulk cytoplasmic Ca and *I*_NCX_ during spontaneous and caffeine-induced Ca release, with a larger current for a given Ca when Ca is increasing than when it is decreasing (35). We have proposed that this is because *I*_NCX_ occurs predominantly at T tubules, where NCX will be exposed to a higher Ca than that in the bulk cytoplasm during Ca release (28). If so, DT would be expected to reduce the hysteresis and CAL to change it because of altered NCX distribution.

**Fig. 3.**
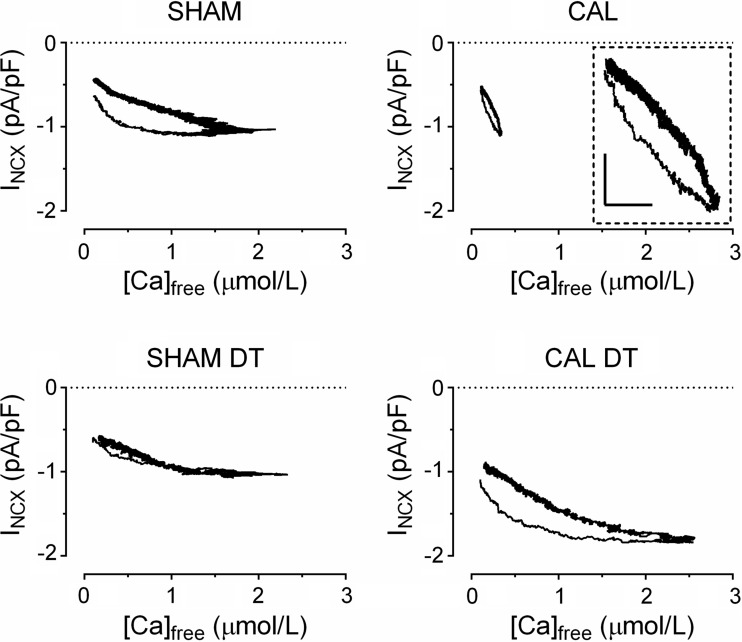
Averaged hysteresis loops for *I*_NCX_ density vs. [Ca]_i_ during application of caffeine in Sham, DT Sham, CAL, and DT CAL myocytes; *n* = 11/5, 9/5, 7/4, 7/4, respectively. *Inset*: loop on expanded scales: *x* and *y*-scale bars represent 0.1 μM and 0.2 pA/pF, respectively.

To test this idea we plotted free [Ca]_i_ against current density during application of caffeine for each of the four groups of cells. Figure 3 shows the average hysteresis loops for each group of cells; each loop consists of the data from all the cells in that group. Figure 3, *left*, shows the relationship in intact (*top*) and DT (bottom) Sham myocytes. Intact cells show a hysteresis, as previously described (35), with more current at a given [Ca]_i_ during the rising phase. However, this hysteresis is reduced in DT myocytes, consistent with idea that the hysteresis arises at the T tubules, because of proximity of NCX to SR Ca release via RyRs.

Figure 3, *right*, shows similar traces obtained from CAL myocytes. The loop obtained in CAL myocytes was smaller than in Sham cells, presumably because the mean rise of free [Ca]_i_ was smaller in these cells (Fig. 1*A*), although the hysteresis was maintained (see *inset*). Interestingly, the hysteresis is still apparent in DT CAL myocytes, suggesting that the hysteresis arises at the surface membrane in contrast to its t-tubular location in Sham myocytes and consistent with redistribution of NCX to the surface membrane in these cells.

## DISCUSSION

The present study shows that in CAL myocytes, NCX activity decreases at the T tubules and increases at the surface membrane. This is similar to the redistribution of *I*_Ca_ that we have previously reported (7) and is associated with changes in the fraction of Ca removed by NCX and SR, the balance of Ca influx versus efflux across the t-tubular membrane, and the relationship between [Ca]_i_ and *I*_NCX_. This is important in understanding the role of the T tubules, and their loss, in Ca handling and the generation of DADs in HF.

### 

#### Redistribution of NCX in CAL myocytes.

*I*_NCX_ decreases at the T tubules and increases at the cell surface of CAL myocytes, with no change in whole cell current. Although *I*_NCX_ depends on Ca at the cytoplasmic face of the exchanger, this redistribution is unlikely to be due to local differences in Ca, because caffeine was used to release total SR Ca content and *I*_NCX_ distribution calculated at a fixed Ca, late during the descending phase of the caffeine transient, when Ca, and thus stimulation of NCX, is likely to be uniform throughout the cell. The redistribution is also unlikely to be due to changes in T tubule density, which is unaltered in this model of HF (7), nor is it likely that a change in surface area-to-volume ratio, which was not significantly different in Sham and CAL myocytes, contributes to the observed changes.

The mechanism of redistribution is unclear, although reminiscent of the redistribution of β_2_-adrenoceptors and *I*_Ca_ from their normal t-tubular location to a more uniform distribution in cells from failing hearts (6, 7, 27). It has been suggested that localization of NCX activity at the T tubules is due, in part, to local protein kinase A activity (10); however, protein kinase A activity appears to increase at the T tubules in CAL myocytes (7), making it unlikely that this can explain the decrease in t-tubular *I*_NCX_ observed in these cells. These changes may reflect reversion in HF toward a more neonatal phenotype, in which cell activation is dependent on Ca influx and efflux across the surface, rather than t-tubular, membrane (11, 31) and a general loss of t-tubular protein localization.

#### Functional consequences of redistribution.

Computer modeling suggests that the relative location of NCX, SERCA, and sarcolemmal Ca ATPase alters their ability to compete for cytoplasmic Ca and thus the amount of Ca removed by each pathway (30). The present work shows that DT of Sham myocytes decreases Ca efflux via NCX, as a result of loss of t-tubular NCX, and increases SR Ca uptake. A similar decrease in Ca efflux via NCX and increased Ca uptake via SERCA occurred in CAL myocytes, compared with Sham cells, even though total NCX density at a given [Ca]_i_ is the same in CAL and Sham myocytes. This can be explained by redistribution of NCX away from the T tubules in CAL cells, so that it no longer has “privileged” access to Ca released from the SR as a result of its proximity to RyRs; this will reduce Ca extrusion via NCX and allow a greater fraction of the cytoplasmic Ca to be removed by SERCA, much of which also appears to be located at T tubules (24) where Ca release occurs. Although a large fractional decrease in Ca extrusion via NCX results in a relatively small fractional increase in SR Ca uptake, this reflects the relatively small fraction of Ca removed by NCX compared with the SR. Altered NCX location in CAL myocytes, which was measured when cytoplasmic Ca was relatively uniformly distributed, is likely to be important during the systolic Ca transient, since NCX activity close to the site of CICR at the T tubules, where the majority of Ca efflux normally occurs (Fig. 1*D*), will be reduced, although this may be offset by reduced Ca release at the T tubules due to decreased t-tubular *I*_Ca_.

It is notable that although fractional SR Ca uptake was greater in CAL than in Sham myocytes (Fig. 2*A*), SR Ca content was not significantly different and the caffeine-induced rise of cytoplasmic Ca was smaller (Fig. 1*A*). However, calculated peak [Ca]_i_ depends on [Ca]_rest_, which was taken as 0.1 μmol/l in the present study; increasing [Ca]_rest_ would increase peak [Ca]_i_, but previous reports of [Ca]_rest_ in CAL have been inconsistent, showing an increase, decrease, or no change (9, 17, 19, 37). Nevertheless, the present observations may be reconciled by increased fractional SR Ca uptake being offset by decreased local *I*_Ca_, which would tend to decrease SR Ca content and the increased Ca buffering observed in CAL myocytes, which would decrease free Ca for a given release. This may also account for the greater slope of the relationship between Ca and *I*_NCX_ in CAL myocytes (Fig. 3), since a given Ca extrusion would result in a smaller change in free Ca. However, DT sufficiently increased SR Ca content to cause a larger caffeine-induced rise of cytoplasmic Ca with hysteresis evident in *I*_NCX_ between the rising and falling phases of Ca release. An alternative explanation for the increased SR Ca uptake is increased SERCA activity in CAL myocytes, although this seems unlikely since previous work has shown decreased SERCA activity in HF (16) and this alone would not explain the lack of effect of DT on the contribution of different efflux pathways to Ca removal.

It is also notable that despite the decreased percent contribution of NCX to Ca removal in CAL myocytes, *k*_NCX_ was not significantly different from that in Sham cells. Thus it appears that NCX can rapidly remove Ca from the cytoplasm in the absence of a functional SR (*k*_NCX_), but its fractional contribution is decreased, presumably because its ability to compete with SERCA is decreased as a result of its relocation.

This redistribution of *I*_NCX_ is also likely to be important because HF is associated with disorganization and loss of T tubules (13, 21–23) and redistribution of *I*_Ca_ (and thus Ca release) from the T tubules to the surface membrane. The present work shows that t-tubular *I*_NCX_/*I*_Ca_ density is higher in CAL myocytes than in Sham, suggesting that loss of T tubules will lead to greater loss of Ca efflux, compared with influx, in CAL myocytes, and thus greater Ca accumulation consistent with the observed effect of DT on SR Ca content in these cells. Thus loss of T tubules in HF may result in increased SR Ca content, which will increase both systolic Ca release and the probability of spontaneous SR Ca release and thus of DADs.

The proximity of the majority of NCX adjacent to RyRs at the T tubules may also be important in the genesis of arrhythmias due to activation of NCX by spontaneous SR Ca release in conditions of Ca overload (28). The hysteresis between bulk cytoplasmic Ca and *I*_NCX_ observed during application of caffeine or during spontaneous Ca release (35; Fig. 3) is consistent with Ca released from SR having privileged access to NCX. The observation that DT of Sham myocytes decreased this hysteresis suggests that it arises at the T tubules as a result of the proximity of the majority of NCX to the site of Ca release in the T tubules. However, this hysteresis was evident in CAL and DT CAL myocytes, so that it appears to be occurring at the surface of these cells. The hysteresis in CAL cells cannot be explained by a change in [Ca]_rest_ altering the calibration of [Ca]_i_, which would alter the *x*-axis gain of the hysteresis loops, but the hysteresis would remain. It seems likely, therefore, that the hysteresis in CAL myocytes is due to the redistribution of *I*_NCX_ to the surface membrane, resulting in enhanced *I*_NCX_ in response to Ca at the cell surface, which itself may be increased by the observed redistribution of *I*_Ca_, even in the apparent absence of changes in RyR distribution (7). Thus it appears that privileged access occurs at the cell surface in CAL myocytes, so that loss of T tubules in HF may not protect against DADs, which may be generated at the cell surface and exacerbated by the increase in SR Ca content that accompanies loss of T tubules in these cells.

#### Conclusions.

These data suggest that the cellular distribution of NCX is altered in CAL myocytes and that this will alter NCX activity both directly, by altering the proximity of NCX to the site of SR Ca release, and indirectly, by increasing SR Ca uptake, both in intact myocytes, by decreasing the ability of NCX to compete with SERCA, and following loss of T tubules, which will result in greater loss of NCX than *I*_Ca_. These changes will alter *I*_NCX_ and thus action potential configuration, Ca balance, and the probability, magnitude and site of DAD generation in HF.

## GRANTS

This work was funded by British Heart Foundation Grants PG/10/91/28644, PG/14/65/31055, and RG/12/10/29802.

## DISCLOSURES

No conflicts of interest, financial or otherwise, are declared by the author(s).

## AUTHOR CONTRIBUTIONS

H.C.G. and S.M.B. performed experiments; H.C.G. analyzed data; H.C.G. prepared figures; H.C.G., S.M.B., A.F.J., and C.H.O. edited and revised manuscript; S.M.B., A.F.J., and C.H.O. interpreted results of experiments; A.F.J. and C.H.O. conception and design of research; A.F.J. and C.H.O. approved final version of manuscript; C.H.O. drafted manuscript.
